# Socioeconomic status, family background and other key factors influence the management of head lice in Norway

**DOI:** 10.1007/s00436-014-3833-9

**Published:** 2014-03-08

**Authors:** Bjørn Arne Rukke, Arnulf Soleng, Heidi Heggen Lindstedt, Preben Ottesen, Tone Birkemoe

**Affiliations:** 1 Department of Pest Control, Norwegian Institute of Public Health, Oslo, Norway; 2 Department of Ecology and Natural Resource Management, Norwegian University of Life Sciences, Ås, Norway

**Keywords:** Head lice, Socioeconomic status, Family background, Checking routines, Knowledge, Costs, Guidelines

## Abstract

How head lice infestations are managed by households is an important but generally neglected issue in head lice research. In the present study, we investigate actions taken against head lice by Norwegian households in association with socioeconomic status, family background, school-related variables and other key factors. Repeat questionnaires distributed to caretakers of the same elementary school children during a 2-year period enabled us to study both previous head lice management and any changes in this management through time. Households from 12 schools spanning the main socioeconomic variation found in Norway participated in the study. All students with active head lice infestation were treated in the four investigated periods. Most caretakers used a thorough head lice checking technique and informed others of own infestation. Checking frequency was low as most children were inspected less than monthly. The best determinant of increased checking frequency and thoroughness was personal experience with head lice. The increased awareness, however, seemed to be somewhat short-lived, as there was a decrease in checking frequency and thoroughness within 1 year after infestation. Personal experience with head lice also increased general knowledge related to the parasite. Parents born in developing countries checked their children for head lice more frequently, although less thoroughly, informed fewer contacts when infested, used pediculicides preventively more often and knew less about head lice than parents born in developed countries. Households with highly educated mothers had a lower checking frequency, but their knowledge and willingness to inform others was high. Single parents were more concerned about economic costs and kept children home from school longer while infested than other parents. As head lice management varied among socioeconomic groups and with parental background, differentiated advice should be considered in the control of head lice. The biannual focus on head lice during the 2 years of investigation increased checking thoroughness, while checking frequency remained unchanged. Based on the results, we suggest new head lice management guidelines for health authorities.

## Introduction

Head lice (*Pediculus capitis* De Geer) are globally prevalent human parasites (Falagas et al. 2008; Toloza et al. 2009) that cause considerable distress to affected children and their families (Hensel 2000; Parison and Canyon 2010; Tebruegge et al. 2011; Parison et al. 2013). In some developed countries, head lice infestations also consume important resources from public health institutions (Jahnke et al. 2008; Rukke et al. 2011). The primary route of head lice transmission is head-to-head contact (Canyon et al. 2002; Mumcuoglu et al. 2009; Heukelbach 2010). To efficiently decrease the prevalence of head lice in a community, all persons or families at risk of being infested should be engaged. If some groups are disinterested in head lice detection and avoid taking actions when infested, the effect of actions taken by others will be reduced as long as there is contact between the groups. Elementary school children generally show the highest prevalence of head lice (Burgess 1995; Roberts 2002; Leung et al. 2005; Rukke et al. 2011), and since students are intermingled in classes and have high contact rates (Mossong et al. 2008), transmission of head lice occurs frequently. Therefore, it is particularly important to provide general knowledge and teach effective routines regarding head lice management to households of school-age children in order to combat head lice in a community.

Studies of head lice have primarily focused on aspects of insect biology, epidemiology and efficacy of pediculicides (Heukelbach 2010). This is important for quantifying and understanding the character of head lice infestation, as well as for developing effective treatments. However, to reduce the prevalence of head lice, it is also important to note what people in a community actually do when they face pediculosis. Such information is remarkably scarce in the literature, though some exceptions exist (Counahan et al. 2007; Heukelbach and Ugbomoiko 2011; Rukke et al. 2012).

Checking routines are essential for suppressing head lice infestations. This includes both checking frequency and thoroughness. A head lice infestation can be asymptomatic or remain undetected for several weeks (Heukelbach and Feldmeier 2010), which influences the length of the infectious period. In Norway, where most people successfully treat pediculosis, these factors might be the primary determinants of infestation time (Rukke et al. 2012). The duration of the infectious period is important to the spread of any directly transmitted parasite (Begon 2009), and regular, thorough head inspections are, therefore, crucial to decreasing the overall prevalence of head lice. When the risk of getting head lice is high, such as during peak incidence seasons in late summer and early autumn in Europe (Bauer et al. 2009) or when close friends or schoolmates are infested, the checking frequency and thoroughness should be intensified (Rukke et al. 2012). This can only be achieved with open communication among peers and families regarding pediculosis (Nutanson et al. 2008; Laguna and Risau-Gusman 2011; Mumcuoglu et al. 2007).

To improve the management of head lice, counselling efforts from public health authorities should aim to reach all persons at risk. Educational material should be appropriate for and ideally matched to the education and reading levels of particular target audiences and be compatible with their ethnic and cultural backgrounds (Resnicow et al. 2002). As with any transfer of information, there is also a need to emphasise the most important messages. However, the most needed information may differ among groups of a population (Glantz et al. 2008), and knowledge of such differences is important and should also be investigated. The goal of head lice education is to decrease the prevalence of infestations, but the educational needs may vary among families of different socioeconomic status (Willems et al. 2005), schools and other subgroupings (Rukke et al. 2012).

In the present study, we have evaluated actions taken against head lice among elementary school children in Norway in relation to socioeconomic status, family background, school-related variables and other factors. To accommodate variations in socioeconomic status, the study was conducted in the city of Oslo, which is representative of the socioeconomic range found in the entire country (Mogstad 2005). We evaluated two datasets: one based on a questionnaire that asked for previous experience and actions taken against head lice, and one based on repeat questionnaires to the same students during two successive school years. Following the same students over time provided a unique opportunity to study changes in actions based on repeated attention and informational campaigns. To assess the impact of pediculosis on the community, indirect and direct costs associated with pediculosis were included in the questionnaires.

## Materials and methods

### Participants

Students from 12 elementary schools (first through seventh grade) in Oslo were invited to participate in the study. The schools had an average size of 472 students (range 337–615). The schools were selected by stratified sampling to represent four geographic and socioeconomic regions in Oslo (Mogstad 2005). Schools were randomly selected within each of these regions.

Caretakers approved the participation of elementary school students through written consent. The Data Protection Agency of Norway, the Regional Committees for Medical and Health Research Ethics in Norway and the owners of various government data sets (the Ministry of Education and Research, the Norwegian Labour and Welfare Administration and the Directorate of Taxes) all approved the use of data from their respective sources for the study.

### Sampling process

Questionnaires were addressed to the parents/caretakers of the participating students. They were distributed at the beginning of the study and at the end of three successive time periods: Start (experience prior to September 2008), period 1 (October 2008 to May 2009), period 2 (June 2009 to November 2009) and period 3 (December 2009 to June 2010). The students received a lice information brochure and a white plastic lice comb (“PDC”, KSL Consulting, Denmark) with the questionnaires.

Predefined categories were used in the questionnaires to elicit information regarding head lice checking routines (frequency and thoroughness), preventive use of pediculicides and direct economic costs and cost concerns regarding pediculosis treatment (i.e. considering not to treat an infestation with pediculicides due to high prices). Head lice checking frequency was categorized as infrequent (less than monthly, only biannually or never) or frequent (monthly or more often), and checking method was categorized as thorough (using a lice comb or a lice comb and fingers) or not thorough (using fingers, ordinary comb or not checking at all). Level of knowledge was established through 13 true or false statements in September 2008 only; it was categorized as high or low based on the total score of correct answers. The participants also reported episodes of pediculosis during the four time periods. If infested, they provided information about the type of treatment used, who they informed about the infestation and whether or not the child had been taken out of school when infested. The data on head lice prevalence gathered in this survey is reported by Birkemoe et al. (unpublished results).

For each student, Statistics Norway provided the following parameters: mother’s and father’s working hours (categorized as short (<30 h per week) or long (≥30 h per week)); mother’s and father’s highest education level (primary school, secondary school or higher education); total household income (categorized as low (<500,000 Norwegian crowns (NOK, 1.0 NOK ≈ €0.125 at the time of the study)), medium (500,000–875,000 NOK) or high (>875,000 NOK); family (ethnic) background (parents’ countries of birth (categorized as Norway, Western countries (North America, Europe and Oceania) or developing countries (Asia, Africa and South America)); the number of siblings living in the same household; student’s sex; the number of caretakers; and the number of children younger than 16 years old living in the household.

### Statistical analyses

Multivariate, mixed-effect (multilevel) logistic regression models were used to analyse the effect that several predictor variables had on different binary response variables (checking frequency, checking thoroughness, preventive pediculicide use, informing others about own pediculosis, cost concerns regarding treatment of pediculosis, keeping children home from school and level of knowledge). Only the predictor variables that in the preliminary univariate logistic analysis had *p* values <0.15 were included in the multivariate models. Multivariate mixed-effect models contain fixed effects and random effects, the latter of which account for a hierarchical structure of data. School was included as a random-effect variable in all models to account for the fact that study units from the same school could be more dependent on each other than study units from other schools. We used univariate logistic regression to analyse changes in certain response variables over the different periods of investigation. Statistical analyses were performed using Stata software version 11 (StataCorp LP 2009).

## Results

### Participation

A total of 5,663 students from 12 schools were invited to participate in the study, and 2,510 students returned the questionnaire in September 2008, which corresponds to a participation rate of 44 % (Table 1). The participation rate dropped in the subsequent investigations. A total of 608 students participated in the entire study and returned all four questionnaires. The differences in participation among schools were consistent over time, and the schools with the highest and lowest participation rates remained the same during the 2-year study period.

**Table 1:** Students participating at the 12 schools in the study

Period of study	% Participation (*n*)	% Participation per school (min-max)
Start (September 2008)	44.3 (5,663)	18.4–62.0
Period 1 (October 2008–June 2009)^a^	35.7 (5,246)	17.3–49.6
Period 2 (July 2009–November 2009)^b^	35.5 (4,930)	15.7–49.2
Period 3 (December 2009–June 2010)	28.2 (4,930)	10.8–47.9

^a^One school chose not to participate

^b^Seventh grade students enrolled in 2008 had left school

### Checking frequency and method

At the start of the study, a minority of the students (37.1 %) had been checked for head lice monthly or more often (Table 2). However, when students were checked, most (69.5 %) were checked thoroughly with a lice comb. The checking frequency did not differ among the four periods investigated, while checking thoroughness significantly improved over the course of the study.

**Table 2:** Univariate, logistic regression models of changes in checking routines (checking frequency (often or rare) and checking thoroughness (thoroughly or not thoroughly)) among all students (two uppermost models) and among students who experienced head lice during the study (two lower models). Odds ratios are in relation to the first category in each model

Model	*p* value	Period	Checking often/thoroughly (*n*)	Odds ratio (95 % CI)
All students				
Checking frequency	0.526	Start	37.1 % (2,486)	1
		Period 1	39.2 % (1,958)	1.09 (0.97–1.24)
		Period 2	38.0 % (1,754)	1.04 (0.92–1.18)
		Period 3	37.6 % (1,438)	1.02 (0.89–1.17)
Checking thoroughness	<0.001	Start	69.5 % (2,373)	1
		Period 1	71.5 % (1,719)	1.10 (0.96–1.26)
		Period 2	74.9 % (1,480)	1.31 (1.13–1.51)
		Period 3	76.2 % (1,216)	1.41 (1.20–1.65)
Students with lice infestation September 2008–June 2010				
Checking frequency	<0.001	Start	38.9 % (180)	1
		Period 3	58.2 % (137)	2.21 (1.40–3.47)
Checking thoroughness	0.024	Start	71.5 % (179)	1
		Period 3	82.4 % (136)	1.86 (1.08–3.21)

#### *Did checking routines change after head lice experience?*

Students who had a head lice infestation once or more during the study period reported improved checking frequency and thoroughness at the end of the study compared to the start (Table 2). Of children who experienced a head lice infestation in period 1, 63.9 % (*n* = 108) were checked monthly or more often within the same time period, whereas only 54.2 % (*n* = 59) and 54.9 % (*n* = 51) of the same students were checked as frequently in periods 2 and 3, respectively. For these students, the rates of checking thoroughness were 87.2 % (*n* = 109), 82.8 % (*n* = 58) and 78.4 % (*n* = 51) for the same three periods. Although the sample size was too low for statistical analysis, the data indicate that increased checking frequency and thoroughness decrease relatively soon following pediculosis.

#### *What triggered caretakers to check their children?*

**Fig. 1:**
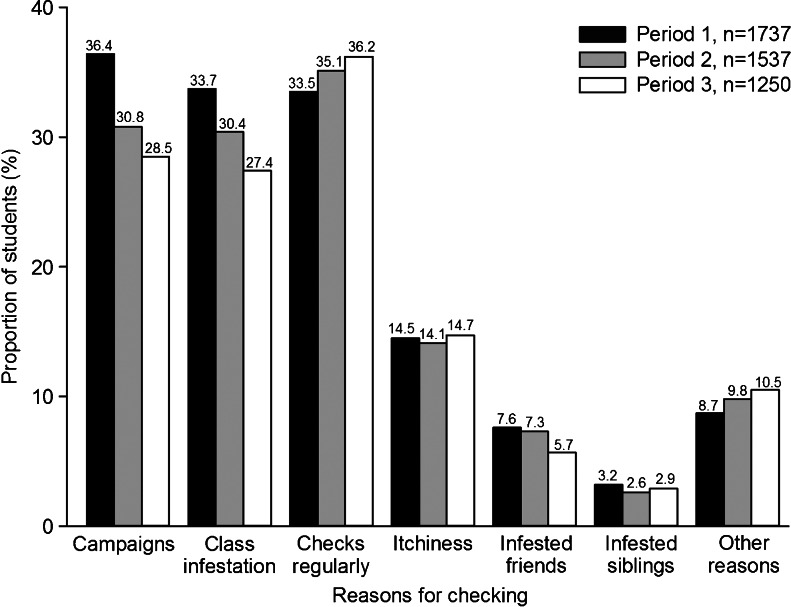
Reasons why households checked their student for head lice during the three investigated periods. The exact percentage is written above each *bar*

When caregivers were asked why the students were checked for pediculosis, the three most common answers in the investigated periods were “because of head lice campaigns”, “reported lice in the class” and “always checks regularly” (Fig. 1).

#### *Who had the best checking routines?*

**Fig. 2:**
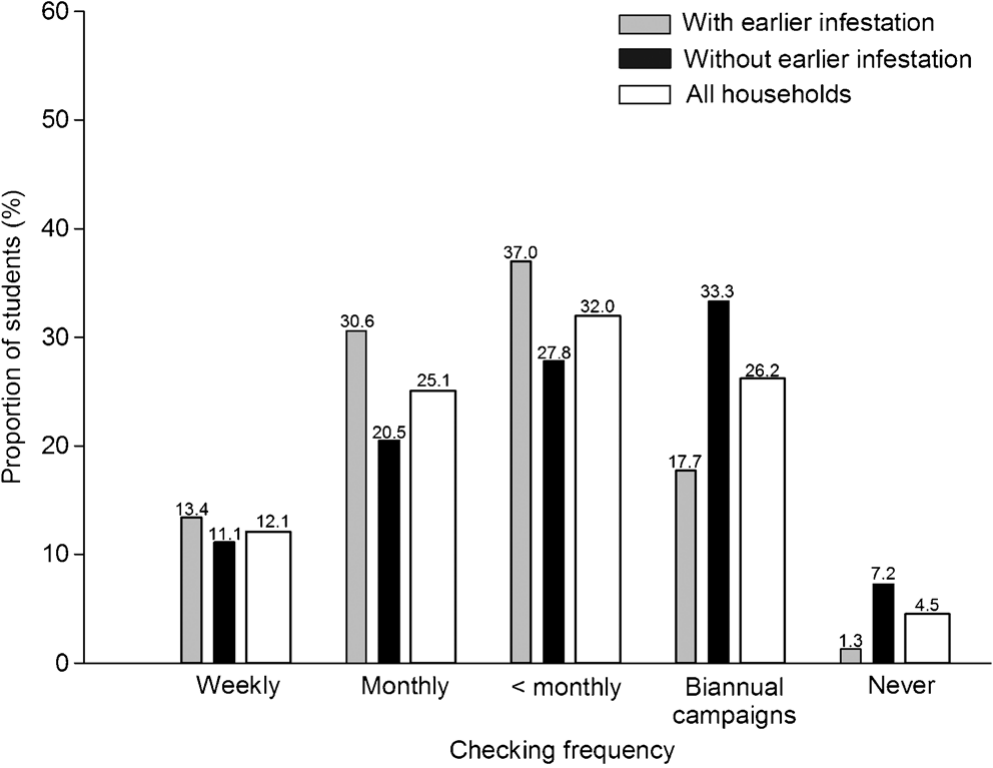
Checking frequencies reported at the start of the investigation in students with and without previous head lice infestations in the household (either the participating child or siblings), as well as in all students combined. The exact percentage is written above each *bar. n* = 2,447

**Fig. 3:**
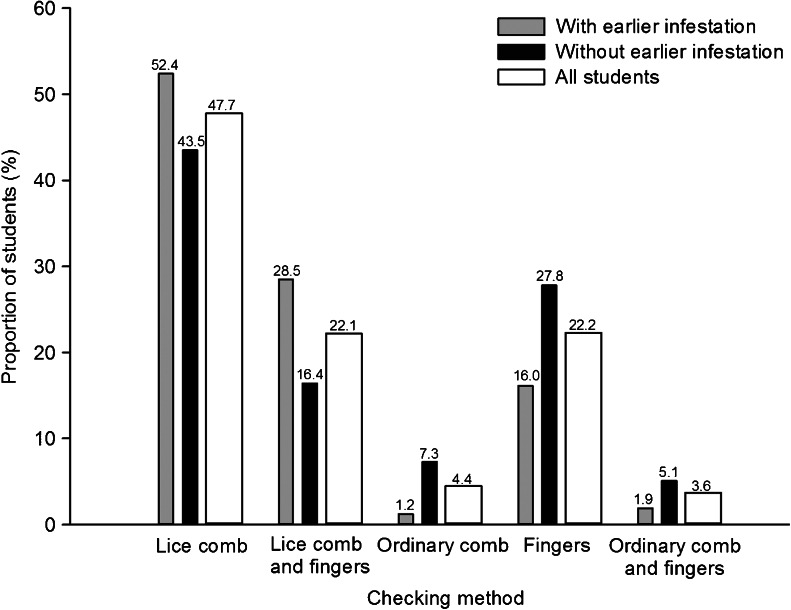
Checking methods reported at the start of the investigation in students with and without previous head lice infestations in the household (either the participating child or siblings), as well as in all students combined. The exact percentage is written above each *bar. n* = 2,291

Data from the start of the investigation showed that children who had experienced head lice in their household in the past (either the participating child or siblings) were checked more frequently and thoroughly than children who had not experienced an infestation in their household (Tables 3 and 4, Figs. 2 and 3). Checking frequency was highest for children in second to fourth grade, while thoroughness was best among those from third to sixth grade. Children with parents from developing countries were checked more often than those with parents born in Norway, but the latter group was checked more thoroughly than those with parents born elsewhere. Children from households with more than three children tended to be checked more often than smaller households, and children from households with only one child were checked less thoroughly than children from other families. Children whose mothers had the highest education level or worked long hours (*p* = 0.065) were checked less frequently than those with less educated mothers or mothers who worked fewer hours. Finally, higher knowledge of head lice increased thoroughness compared to lower knowledge. School, the random-effect variable, significantly improved both models of checking routines (estimate for frequency 0.377, *p* < 0.001; estimate for thoroughness 0.566, *p* < 0.001).

**Table 3:** Multivariate, mixed-effect logistic regression model of checking frequency (often or rare) in students with school as a random-effect variable. All data were reported at the start of the investigation. Odds ratios are in relation to the first category of each variable

Variable	*p* value	Category	Checking often (*n*)	Odds ratio (95 % CI)
Previous occurrence of head lice in household	<0.001	No	31.6 % (1,340)	1
		Yes	44.0 % (1,107)	1.74 (1.44–2.09)
Grade	<0.001	1.	33.6 % (428)	1
		2.	42.5 % (358)	1.44 (1.06–1.95)
		3.	46.4 % (397)	1.58 (1.17–2.12)
		4.	46.7 % (362)	1.55 (1.14–2.10)
		5.	31.8 % (352)	0.73 (0.53–1.01)
		6.	27.7 % (271)	0.60 (0.42–0.86)
		7.	26.9 % (279)	0.62 (0.44–0.88)
Family background	0.041	Norway	35.1 % (1,679)	1
		Western	38.5 % (340)	1.07 (0.82–1.39)
		Developing	44.6 % (428)	1.42 (1.08–1.87)
Children (<16 years)	0.077	1	35.9 % (526)	1
		2	35.1 % (1,275)	0.93 (0.73–1.18)
		3	39.6 % (533)	1.03 (0.77–1.37)
		>4	55.8 % (113)	1.64 (1.03–2.59)
Parents	0.359	1	39.9 % (411)	1
		>1	36.7 % (2,036)	0.89 (0.69–1.15)
Education of mother	0.002	Primary	40.9 % (465)	1
		Secondary	39.0 % (687)	0.83 (0.61–1.12)
		Higher	35.0 % (1,295)	0.61 (0.45–0.83)
Education of father	0.058	Primary	40.9 % (465)	1
		Secondary	39.0 % (687)	1.20 (0.91–1.59)
		Higher	35.0 % (1,295)	0.92 (0.70–1.21)
Working hours of mother	0.066	Short	42.8 % (825)	1
		Long	34.4 % (1,622)	0.83 (0.69–1.01)
Working hours of father	0.441	Short	41.1 % (445)	1
		Long	36.4 % (2,002)	0.91 (0.72–1.16)
Checking thoroughness	0.147	Not thorough	32.4 % (818)	1
		Thorough	39.7 % (1,629)	1.16 (0.95–1.42)

**Table 4:** Multivariate, mixed-effect logistic regression model of checking thoroughness (thorough or not thorough) in students with school as a random-effect variable. All data were reported at the start of the investigation. Odds ratios are in relation to the first category of each variable

Variable	*p* value	Category	Checking thoroughly (*n*)	Odds ratio (95 % CI)
Previous occurrence of head lice in household	<0.001	No	55.6 % (1,307)	1
		Yes	80.2 % (1,088)	2.56 (2.08–3.15)
Grade	0.001	1.	57.5 % (421)	1
		2.	63.1 % (352)	1.12 (0.81–1.54)
		3.	70.0 % (387)	1.50 (1.08–2.07)
		4.	72.4 % (359)	1.87 (1.33–2.62)
		5.	68.4 % (345)	1.34 (0.96–1.87)
		6.	72.0 % (261)	1.94 (1.34–2.83)
		7.	66.3 % (270)	1.33 (0.93–1.90)
Family background	<0.001	Norway	71.6 % (1,659)	1
		Western	60.5 % (332)	0.58 (0.44–0.77)
		Developing	51.7 % (404)	0.61 (0.46–0.82)
Children (<16 years)	<0.001	1	59.7 % (514)	1
		2	66.8 % (1,254)	1.35 (1.05–1.73)
		3	72.8 % (522)	1.77 (1.29–2.41)
		>4	70.5 % (105)	2.72 (1.60–4.61)
Parents	0.630	1	62.3 % (398)	1
		>1	67.6 % (1,997)	1.07 (0.81–1.41)
Sex	0.514	Male	65.1 % (1,148)	1.06 (0.88–1.29)
		Female	68.2 % (1,247)	
Education of mother	0.750	Primary	52.7 % (347)	1
		Secondary	62.6.% (610)	1.04 (0.76–1.43)
		Higher	71.8 % (1,438)	1.12 (0.81–1.53)
Education of father	0.588	Primary	58.2 % (447)	1
		Secondary	62.7 % (675)	0.90 (0.68–1.20)
		Higher	71.9 % (1,273)	1.02 (0.77–1.36)
Knowledge	<0.001	Low	55.0 % (906)	1
		High	73.9 % (1,489)	1.55 (1.26–1.90)
Checking frequency	0.177	Rare	64.1 % (1,505)	1
		Often	71.2 % (890)	1.15 (0.94–1.42)

### Treatment method

**Fig. 4:**
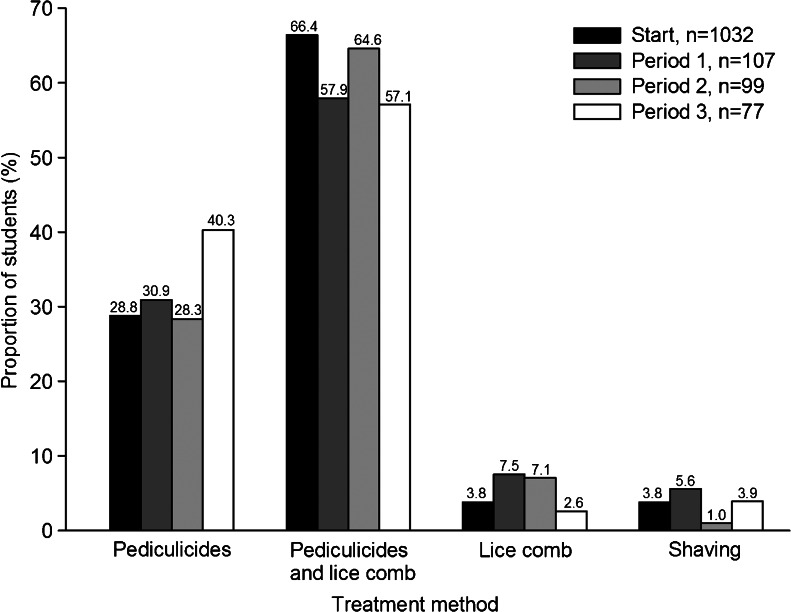
Head lice treatment methods for infested students during the four investigated periods. The exact percentage is written above each *bar*

**Fig. 5:**
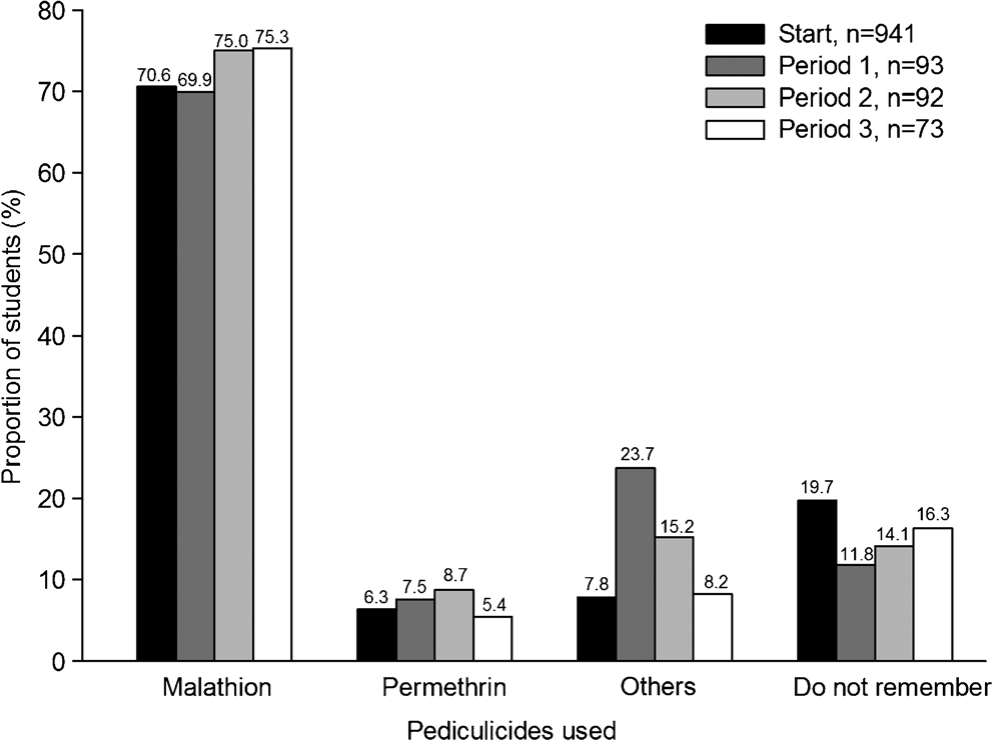
Pediculicide products used by infested students during the four investigated periods. The exact percentage is written above each *bar*

Nearly all students (99.7 %) with a previous infestation at the start of the investigation and all infested students in the following periods were treated for head lice. More than 88 % were treated with pediculicides, usually in combination with a lice comb (Fig. 4). Less than 10 % used a lice comb as the sole method of treatment, and fewer than 6 % reported head shaving as a treatment (Fig. 4). The most common choices of pediculicides were products containing malathion (Fig. 5).

### Preventive use of pediculicides

At the start of the investigation, 8.8 % of the students (*n* = 2,406) had been treated preventively with pediculicides. Of these, 54.7 % had been treated because siblings were infested. Other reasons for preventive pediculicide treatment included information about pediculosis distributed at school (16.0 %), pediculosis among friends (20.8 %) and/or information from the school about current infestations in the class or school (20.8 %).

#### *Who used pediculicides preventively?*

The likelihood of a student being preventively treated with pediculicides increased with the number of siblings, parental origin outside Norway, previous pediculosis in the household, thorough head lice checking routines and poor head lice knowledge within the family (Table 5). School affiliation significantly affected the preventive use of pediculicides (estimate 0.334, *p* = 0.020).

**Table 5:** Multivariate, mixed-effect logistic regression model of preventive use of pediculicides (used or not used) in students with school as a random-effect variable. All data were reported at the start of the investigation. Odds ratios are in relation to the first category of each variable

Variable	*p* value	Category	Used preventively (*n*)	Odds ratio (95 % CI)
Previous occurrence of head lice in household	<0.001	No	6.0 % (1,247)	1
		Yes	11.8 % (1,083)	1.94 (1.39–2.70)
Grade	0.214	1.	4.7 % (404)	1
		2.	7.8 % (346)	1.55 (0.83–2.88)
		3.	8.5 % (376)	1.68 (0.92–3.07)
		4.	9.5 % (347)	1.64 (0.89–3.01)
		5.	9.8 % (338)	1.87 (1.02–3.43)
		6.	11.9 % (252)	2.19 (1.17–4.09)
		7.	10.8 % (267)	2.11 (1.13–3.94)
Family background	0.001	Norway	6.6 % (1,634)	1
		Western	12.5 % (321)	1.93 (1.28–2.90)
		Developing	14.9 % (375)	1.98 (1.25–3.11)
Children (<16 years)	0.003	1	6.6 % (502)	1
		2	7.7 % (1,229)	1.36 (0.88–2.09)
		3	10.7 % (505)	1.73 (1.07–2.79)
		>4	23.4 % (94)	3.38 (1.74–6.58)
Sex	0.088	Male	7.3 % (1,107)	1
		Female	10.0 % (1,223)	1.30 (0.96–1.77)
Education of mother	0.724	Primary	14.3 % (335)	1
		Secondary	8.1 % (584)	0.84 (0.50–1.39)
		Higher	7.7 % (1,411)	0.82 (0.49–1.36)
Education of father	0.308	Primary	12.1 % (431)	1
		Secondary	9.0 % (646)	1.06 (0.67–1.67)
		Higher	7.4 % (1,253)	0.79 (0.50–1.24)
Working hours of father	0.296	Short	12.7 % (403)	1
		Long	7.9 % (1,927)	0.81 (0.55–1.19)
Knowledge	0.023	Low	11.1 % (859)	1
		High	7.3 % (1,471)	0.67 (0.47–0.94)
Checking thoroughness	0.047	Not thorough	6.4 % (766)	1
		Thorough	9.9 % (1,564)	1.44 (1.00–2.09)

### Informing others about own infestation

**Fig. 6:**
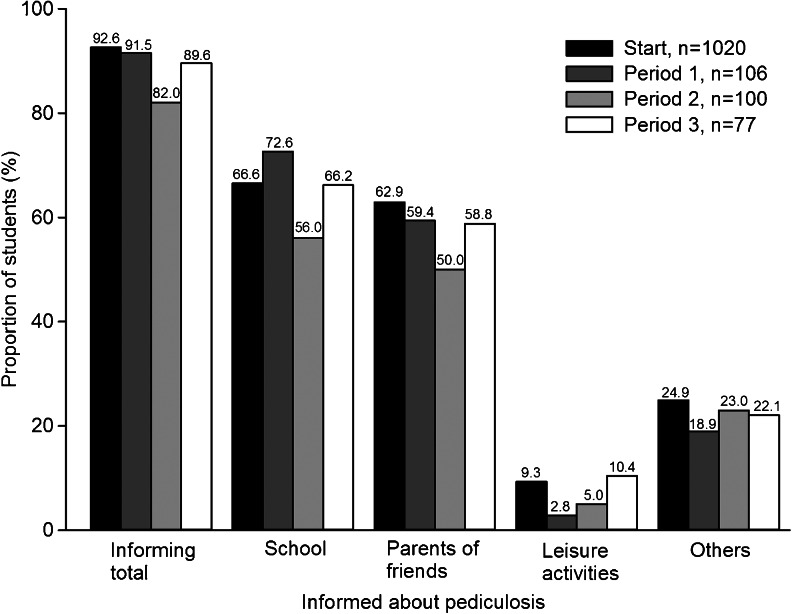
Groups informed when students had head lice during the investigation. The exact percentage is written above each *bar*

In all investigated periods, the majority of households reported that they had informed others about their child’s pediculosis (Fig. 6). Approximately two thirds of the families informed the school or parents of friends, while substantially fewer informed contacts from leisure activities or others.

#### *Who informed less frequently?*

Parents born in developing countries informed others less frequently than those with parents from other countries (Table 6). Also, parents with little formal education and low knowledge about head lice informed others less often than other parents. School affiliation did not significantly affect the willingness to tell others about an infestation within one’s own household (estimate 0.240, *p* = 0.276).

**Table 6:** Multivariate, mixed-effect logistic regression model of informing others about the student’s pediculosis (informing or not informing) with school as a random-effect variable. All data were reported at the start of the investigation. Odds ratios are in relation to the first category of each variable

Variable	*p* value	Category	Informing others (*n*)	Odds ratio (95 % CI)
Family background	0.001	Norway	96.4 % (723)	1
		Western	92.4 % (144)	0.60 (0.27–1.31)
		Developing	74.5 % (137)	0.25 (0.12–0.50)
Children (<16 years)	0.236	1	94.9 % (214)	1
		2	93.4 % (514)	0.61 (0.28–1.34)
		3	93.2 % (235)	0.70 (0.28–1.74)
		>4	73.2 % (41)	0.32 (0.11–0.97)
Parents	0.153	1	91.5 % (176)	1
		>1	93.1 % (828)	1.71 (0.83–3.52)
Education of mother	0.015	Primary	73.5 % (117)	1
		Secondary	93.5 % (245)	2.28 (1.07–4.88)
		Higher	96.1 % (642)	3.26 (1.46–7.28)
Education of father	0.533	Primary	84.1 % (157)	1
		Secondary	93.3 % (282)	1.41 (0.66–3.00)
		Higher	95.0 % (565)	0.97 (0.45–2.09)
Working hours of mother	0.207	Short	88.5 % (348)	1
		Long	95.1 % (656)	1.44 (0.82–2.53)
Working hours of father	0.366	Short	88.4 % (155)	1
		Long	93.6 % (849)	0.72 (0.35–1.48)
Knowledge	0.035	Low	83.6 % (256)	1
		High	96.0 % (748)	1.97 (1.06–3.66)

### Costs

The direct cost of pediculosis (i.e. the money spent on lice combs and pediculicides in the previous year) was low when reported at the start of the investigation. More than half of the households (58.3 %, *n* = 2,074) spent less than 50 NOK and only 2 % spent more than 1,000 NOK. Inside those extremes, 13.6 % spent between 250 and 1,000 NOK and 25.7 % spent between 50 and 250 NOK. Only 7.6 % (*n* = 961) of the households reported finding costs of pediculicides so high that they had not treated or considered not treating their infested child.

Approximately one in three children were kept home from school during a head lice infestation prior to the start of the study (30.9 %, *n* = 1,018). Through the next three investigated periods, 21.6 % (*n* = 255) were kept home from school during an infestation. Of these, 26.9 % (*n* = 67) were kept home less than 1 day, 68.7 % 1 or 2 days and 4.5 % more than 2 days.

#### *Who were most concerned about costs and kept children home from school?*

Families that spent considerable money on pediculosis remedies (pediculicides, combs, etc.) in the previous year and single-parent households were more concerned about the costs associated with head lice treatment than other households (Table 7). School affiliation affected concerns about costs (estimate 0.487, *p* = 0.049).

**Table 7:** Multivariate, mixed-effect logistic regression model of the concern of costs regarding pediculicides (considered not to treat or never considered not to treat) in the household of the student with school as a random-effect variable. All data were reported at the start of the investigation. Odds ratios are in relation to the first category of each variable

Variable	*p* value	Category	Considered not to treat (*n*)	Odds ratio (95 % CI)
Money spent on head lice remedies last year	<0.001	<50 NOK	1.8 % (279)	1
		50–250 NOK	4.8 % (315)	1.95 (0.67–5.68)
		250–1,000 NOK	14.6 % (239)	7.68 (2.82–20.94)
		>1,000 NOK	24.4 % (41)	13.84 (4.11–46.58)
Grade	0.596	1.	7.5 % (80)	1
		2.	7.8 % (102)	1.44 (0.43–4.76)
		3.	10.8 % (148)	1.49 (0.50–4.42)
		4.	7.8 % (154)	0.99 (0.32–3.01)
		5.	8.8 % (159)	1.50 (0.50–4.52)
		6.	4.8 % (126)	0.81 (0.23–2.91)
		7.	2.9 % (105)	0.53 (0.11–2.42)
Family background	0.833	Norway	6.6 % (648)	1
		Western	8,7 % (127)	1.27 (0.59–2.74)
		Developing	11.1 % (99)	1.09 (0.44–2.66)
Sex	0.324	Male	5.7 % (368)	1
		Female	8.7 % (506)	1.34 (0.75–2.41)
Parents	0.043	1	12.6 % (151)	1
		>1	6.4 % (723)	0.50 (0.26–0.96)
Education of mother	0.103	Primary	18.6 % (86)	1
		Secondary	8.6 % (210)	0.54 (0.23–1.28)
		Higher	5.4 % (578)	0.39 (0.17–0.91)
Working hours of mother	0.668	Short	10.1 % (288)	1
		Long	6.1 % (586)	0.88 (0.49–1.58)
Working hours of father	0.625	Short	11.3 % (133)	1
		Long	6.8 % (741)	0.84 (0.41–1.71)
Knowledge	0.158	Low	10.6 % (198)	1
		High	6.5 % (676)	0.61 (0.31–1.19)
Checking frequency	0.713	Rare	5.9 % (488)	1
		Often	9.3 % (386)	1.11 (0.62–1.99)

Among the students who were infested with head lice, female students, children with single parents and children with more than one previous infestation of head lice stayed home from school more often than others (Table 8). The school affiliation did not affect the likelihood of keeping a child at home (estimate 0.121, *p* = 0.263).

**Table 8:** Multivariate, mixed-effect logistic regression model of children being kept at home during pediculosis (have been retained or have not been retained) with school as a random-effect variable. All data were reported at the start of the investigation. Odds ratios are in relation to the first category of each variable

Variable	*p* value	Category	Retained children from school (*n*)	Odds ratio (95 % CI)
Sex	0.048	Male	26.9 % (431)	1
		Female	33.9 % (566)	1.33 (1.00–1.76)
Parents	0.004	1	43.8 % (178)	1
		>1	28.1 % (819)	0.59 (0.42–0.84)
Previous infestations in student	0.001	Once	25.6 % (633)	1
		Twice	39.7 % (247)	1.79 (1.30–2.46)
		Three times	38.7 % (75)	1.73 (1.04–2.88)
		>Three times	45.2 % (42)	2.23 (1.16–4.26)
Education of father	0.183	Primary	34.8 % (158)	1
		Secondary	35.0 % (280)	1.05 (0.69–1.60)
		Higher	27.7 % (559)	0.79 (0.53–1.18)
Checking frequency	0.279	Rare	28.7 % (558)	1
		Often	33.7 % (439)	1.17 (0.88–1.54)

### Knowledge

Only 2.3 % of the students’ caretakers responded correctly to all true-false statements regarding head lice (*n* = 2,419), while 74.6 % answered more than half of them correctly. Nearly all (90 % or more) correctly answered that head lice crawl from head to head, that head lice will survive ordinary shampooing and that untreated persons with head lice may repeatedly infest others (Table 9). More than half of the respondents answered incorrectly or responded “do not know” to the statements that head lice can survive several days on clothes or furniture, that some pediculicides kill all eggs, that a home with head lice among its inhabitants must be thoroughly cleaned and that head lice easily spread from pillows, furniture, plush animals and clothes.

**Table 9:** Statements regarding head lice considered by the households at the start of the investigation. The proportion of correct, wrong and “do not know” responses of each statement are listed

**Statement**	Responses (%)		
	Correct	Wrong	Do not know
1. Head lice can jump (False) (*n* = 2,458)	66.9	22.5	10.6
2. Head lice can survive several days on clothes or furniture (False) (*n* = 2,459)	47.4	39.3	13.3
3. Head lice can survive 20 min in a sauna (approx. 80 °C) (True) (*n* = 2,439)	35.1	15.8	49.1
4. Head lice crawl from head to head in close contact (True) (*n* = 2,463)	96.3	1.5	2.2
5. People who get head lice always start to itch immediately (False) (*n* = 2,464)	68.1	21.6	10.4
6. Head lice will survive an ordinary shampooing (True) (*n* = 2,470)	89.8	7.9	2.3
7. Some available pediculicides kill all lice eggs (False) (*n* = 2,449)	33.7	35.8	30.5
8. Only persons having head lice should be treated with pediculicides (True) (*n* = 2,457)	79.4	13.6	7.0
9. The home must be thoroughly cleaned if head lice are found (False) (*n* = 2,442)	49.0	40.2	10.8
10. Head lice can spread from pets or farm animals (False) (*n* = 2,456)	58.6	13.6	27.8
11. Head lice spread easily from pillows, furniture, plush animals and clothes (False) (*n* = 2,458)	33.7	59.1	7.2
12. Treatment with pediculicides must be done twice, 8–10 days apart (True) (*n* = 2,457)	73.5	3.5	23.1
13. Persons having head lice and who are not treated may infest others repeatedly (True) (*n* = 2,466)	96.3	0.6	3.1

#### *Who knew most?*

Households with parents of Norwegian background had a higher level of knowledge regarding head lice than parents from other countries (Table 10). A higher level of knowledge was also associated with previous direct contact with pediculosis (either through the participating child’s or siblings’ infestations), mothers with higher levels of education and checking thoroughness. School affiliation did not affect knowledge (estimate 0.104, *p* = 0.217).

**Table 10:** Multivariate, mixed-effect logistic regression model of level of knowledge among students’ households (high or low level of knowledge) with school as a random-effect variable. All data were reported at the start of the investigation. Odds ratios are in relation to the first category of each variable

Variable	*p* value	Category	High level of knowledge (*n*)	Odds ratio (95 % CI)
Previous occurrence ofhead lice in household	<0.001	No	52.1 % (1,317)	1
		Yes	74.1 % (1,102)	2.36 (1.94–2.89)
Grade	0.071	1.	56.1 % (424)	1
		2.	61.0 % (354)	1.03 (0.75–1.41)
		3.	68.2 % (390)	1.45 (1.05–2.00)
		4.	59.1 % (364)	0.88 (0.64–1.22)
		5.	68.2 % (349)	1.31 (0.94–1.83)
		6.	62.9 % (265)	1.07 (0.75–1.54)
		7.	60.8 % (273)	1.04 (0.73–1.48)
Family background	<0.001	Norway	70.8 % (1,672)	1
		Western	60.7 % (336)	0.78 (0.59–1.01)
		Developing	28.2 % (411)	0.29 (0.22–0.38)
Children (<16 years)	0.674	1	61.5 % (519)	1
		2	64.8 % (1,266)	0.98 (0.77–1.26)
		3	61.4 % (529)	0.89 (0.65–1.20)
		>4	37.1 % (105)	0.63 (0.37–1.08)
Parents	0.984	1	58.9 % (404)	1
		>1	62.8 % (2,015)	1.00 (0.76–1.32)
Education of mother	<0.001	Primary	26.3 % (354)	1
		Secondary	61.3 % (615)	2.84 (2.05–3.93)
		Higher	71.2 % (1,450)	3.42 (2.48–4.70)
Education of father	0.054	Primary	44.9 % (454)	1
		Secondary	58.3 % (679)	0.87 (0.65–1.17)
		Higher	70.2 % (1,286)	1.16 (0.87–1.54)
Working hours of mother	0.300	Short	54.9 % (801)	1
		Long	65.7 % (1,618)	1.11 (0.91–1.37)
Working hours of father	0.484	Short	50.8 % (425)	1
		Long	64.5 % (1,994)	1.10 (0.85–1.42)
Checking thoroughness	<0.001	Not thorough	48.7 % (807)	1
		Thorough	68.9 % (1,602)	1.55 (1.27–1.90)

## Discussion

### Overall significance of actions, costs and knowledge

The majority of the participants followed many of the head lice recommendations provided by the health authorities in Norway. They used a lice comb when checking for head lice, informed others of infestations in their own households and treated active infestations using the recommended pediculicides, often in combination with a lice comb. However, most students were checked for head lice less frequently than the recommended monthly inspections, and such infrequent examinations may give too long infectious periods to prevent the spread of head lice among students.

We were pleased to see that few students used pediculicides preventively, and the direct costs associated with pediculosis were generally low. Still, the expense of pediculicides may be a limitation for some families, especially those suffering repeated infestations. Indirect costs seemed to pose a larger burden than direct costs, partly owing to the fact that one in three children had remained home from school at least 1 day when infested. Furthermore, a majority of caretakers erroneously believed that unnecessary, time consuming and thorough house cleaning was necessary to fight pediculosis and that head lice spread easily through fomites.

All general results were consistent among the different periods investigated, and they also agree with the findings of Rukke et al. (2012) that investigated actions, costs and knowledge of head lice in five geographically separated areas of Norway.

### Knowledge

A higher level of knowledge about public health issues may, as seen for smoking (Lewit et al. 1981; Schneider et al. 1981), or may not, as seen for alcohol consumption and physical inactivity, improve health status (Kenkel 1991). In our study, more knowledge was associated with more thorough checking, less preventive use of pediculicides and more open communication about a household’s own infestation, but it did not improve checking frequency or reduce the proportion of parents staying at home when their children were infested. It is important to be aware of these differences when deciding what kind of measures to implement. Specifically, checking frequency is unlikely to rise with knowledge, and other approaches to learning are needed to change this action.

The level of knowledge was not uniformly distributed among households of different origins or different socioeconomic groups. It was substantially lower among parents from developing countries compared to those with other backgrounds. There may be several reasons for this finding. First, the former group may have difficulties understanding written or oral information in Norwegian. A generally lower degree of adult literacy among those originating from developing countries (CIA 2013) may further support such a trend. Health communication can also be less effective for individuals of lower socioeconomic status, because such information has often been designed by well-educated people for well-educated audiences (Stroebe 2000).

Education, in general, is correlated with the willingness and ability to acquire new knowledge, so it was not surprising to find that higher educated mothers were more knowledgeable about head lice than those with less education. Many studies have shown that knowledge about a large variety of topics is positively related to the socioeconomic status of recipients and, especially, to their level of education (Tichenor et al. 1970; Gaziano 1983; Weenig and Midden 1997).

The most important factor affecting knowledge, however, was previous head lice experience within the family. This illustrates a desire to learn more about the parasite after direct experience. Experience is also considered an efficient way of learning. As introduced by Dewey (1933), the concept of “learning by doing” emphasises that a learning process is generally better when a person has first-hand experience with the problem.

### Checking routines

Rukke et al. (2012) reported that the best instructor of checking routines was learning by experience. The present study revealed the same trend; increased checking frequency and thoroughness were associated with personal head lice experience. The increased awareness, however, was somewhat short-lived, as there was a decrease in checking frequency and thoroughness within 1 year after infestation.

The improved and subsequent decreased checking frequency with increasing school grade might also be explained by personal experience with head lice, since the prevalence of infestation generally increases up to third or fourth grade before levelling off (Rukke et al. 2011; Birkemoe et al., unpublished results). The improvement in checking thoroughness from the first to higher grades indicates that the importance of using a lice comb in inspections is learned and sustained after the first year in elementary school. Further, there is a lower prevalence of head lice in kindergartens than in elementary schools (Rukke et al. 2011). Consequently, fewer kindergarten parents will learn thorough checking routines by experience than elementary school parents.

Family characteristics influenced checking routines. Checking frequency was highest in children with parents from developing countries. This might imply that louse checking is a more natural part of the daily routine, possibly due to a higher prevalence of head lice in the parents’ native countries. In general, the reported prevalence of head lice infestation is higher outside than inside Europe (Heukelbach et al. 2010). At the same time, the lack of use of a lice comb in these families may indicate less knowledge of a lice comb’s superior efficiency compared to other methods of visual inspection (Burgess 1995; De Maeseneer et al. 2000; Mumcuoglu et al. 2001; Balcioglu et al. 2008; Jahnke et al. 2009) or, simply, lower buying power in this group (Bhuller and Brandsås 2013). The more frequent and thorough checking in families with multiple children, which was also reported by Rukke et al. (2012), may be explained by a higher risk of encountering head lice within one’s own household (Rukke et al. 2011), among friends or at school. Interestingly, the observed lower checking frequency among mothers with high education and long working hours may indicate less time available or lower priority among their households for this activity, despite possessing adequate knowledge.

### Preventive use of pediculicides

Use of pediculicides without identifying the presence of head lice should be avoided (Pollack et al. 2000). The rate of such preventive use was similar in our study to that reported in Rukke et al. (2012), but half of what was observed in an Australian study (Counahan et al. 2007). The observed higher preventive pediculicide use among parents with foreign backgrounds might indicate a greater eagerness to treat. Previous infestations in the household also increased the likelihood of preventive use. This may be due in part to the treatment of all children in a family when one was infested. However, it is also possible that earlier experience decreased scepticism of pediculicides or increased the fear of reinfestation, resulting in more preventive use (Rukke et al. 2012). The negative correlation between preventive use and knowledge indicates that increased information can decrease this unwanted action.

### Information

Because most households informed others when their child was infested, close contacts were often enabled to perform inspection and, if necessary, treat infestations. Households with parents from developing countries and parents with low education levels or little general knowledge of head lice were the least likely to inform others about an infestation. The lack of providing information might be due to the fear of being socially stigmatized (Maunder 1985) or a general problem communicating in Norwegian. Also, some parents from developing countries may view pediculosis as a less serious health issue compared to other more serious concerns experienced in their native country or in Norway. For example, mental health problems, infectious diseases and lifestyle- and diet-related disorders are more common among immigrants than natives in Norway (Abebe 2010). A prominent difference also exists between developing countries and developed market economies owing to the fact that, in the former, emotional reactions to head lice are far less significant (Heukelbach and Speare 2010).

### Economic consequences of head lice

The expense of commercial treatment products for a head lice infestation may be a limitation for some families in industrialized countries (Hansen and O’Haver 2004; Parison et al. 2008). In the present study, among those that reported spending considerable money on pediculosis remedies in the previous year, a large proportion found pediculicides so expensive that they considered not using them. This indicates that a particularly high expenditure can reduce the probability of proper treatment of infestations. As reported by Rukke et al. (2012), single-parent households seemed to suffer higher direct and indirect economic burdens of pediculosis than others. In this group, the ability to pay for pediculicides appeared lower and more work hours were lost when infested children were kept home from school.

### School affiliation

Schools are important arenas for the transfer of information, and any differences in addressing head lice management among them should be identified. Similar to Rukke et al. (2012), checking frequency and method differed among schools in our study. Thus, schools in Norway seem to have the potential to influence checking practices and improve household routines through enhanced information transfer. As schools often urge students to participate in biannual head lice campaigns, they may also play an important role in large-scale synchronization of head lice inspection and treatment. Nearly one third of the households in our study stated that this was a reason for checking their children for head lice.

School affiliation also influenced preventive pediculicide use and the view of costs of pediculicides as high. Such trends were not observed in the nationwide study conducted by Rukke et al. (2012), which may be a result of the larger socioeconomic span between schools in the present investigation (Mogstad 2005). In both studies, the decision to keep infested children away from school did not differ among schools. This is positive, since there is a general consensus that students should not stay home from school when experiencing a head lice infestation (Roberts 2002). Thus, this action seems to be decided by parents alone and not influenced by the schools.

### Effect of increased focus on head lice in schools

An intense health education program had a positive effect on knowledge, attitudes, practice and infestation rate in an investigation of two Iranian schools with high initial prevalence of head lice (Shirvani et al. 2013). In the present study, we hypothesized that a biannual campaign that distributed information, lice combs and questionnaires would lead to increased awareness among the participating households and, consequently, improved management of head lice. Indeed, this seemed true for checking thoroughness, as more caregivers did check their children with a lice comb at the end of the investigation compared to the start. However, for checking frequency no such trend appeared. Thus, there is certainly the potential for learning, but the biannual approach used here is not an effective method for improving all actions related to head lice in a low-prevalence setting in Norway.

### Implementing results in future guidelines

This study underscores several possible improvements that could reduce the prevalence of head lice among Norwegian elementary school students and their households. Actions taken by families varied among schools, socioeconomic groups and family background, and therefore, some issues should be addressed differently to different groups. The following considerations should be included in future guidelines:

- Health authorities should provide updated basic head lice information. Checking frequency, thoroughness and open communication should be emphasised. The importance of more frequent inspections should be highlighted when addressing parents with Norwegian backgrounds, and the importance of thorough checking and openness should be highlighted when addressing parents from developing countries.
- Knowledge about head lice should be increased. This could improve several elements of the management of head lice infestations. Suitable written documents could be supplemented with other sources, such as instructional videos in several languages to increase outreach to families from developing countries and parents with difficulties understanding written information. Preventive information could be included in already existing health care routines, such as visits to public health centres for infants and meetings with the health visitor at schools. There is a large increase in the prevalence of head lice between kindergarten and elementary school (Rukke et al. 2012), so information about head lice should be included in elementary schools’ initial orientation program. Also, recent advances in communication and information technologies involving internet resources allow for a range of strategies for different health change programs (Glantz et al. 2008) and should be utilized in head lice interventions.
- National head lice campaigns like those conducted biannually in Norway should be maintained and supported by national health authorities. Such campaigns have educational benefits, as indicated by checking thoroughness in the present study, and they synchronize inspections on a large scale. Efforts should be taken to ensure that all schools join the campaigns and distribute information efficiently to the students. Handing out louse combs for free during campaigns should be considered. This may increase thoroughness in many households and, possibly, improve the frequency of inspections. Schools in areas where the socioeconomic status is low should be prioritized.
- Health authorities should strive to diminish unnecessary, negative emotions (Parison et al. 2013) and reduce the work load of families affected by pediculosis. Households should be informed that infestations happen irrespective of socioeconomic status and ethnic background. Other factors that should be highlighted include the fact that excessive house cleaning is to be avoided and that children should not be kept home from school due to head lice. If widely distributed and accepted, these and other instructions can simplify pediculosis and ease the burden of experiencing an infestation.
